# Genome-Wide Identification of the *TIFY* Gene Family in *Brassiceae* and Its Potential Association with Heavy Metal Stress in Rapeseed

**DOI:** 10.3390/plants11050667

**Published:** 2022-02-28

**Authors:** Fujun Sun, Zhiyou Chen, Qianwei Zhang, Yuanyuan Wan, Ran Hu, Shulin Shen, Si Chen, Nengwen Yin, Yunshan Tang, Ying Liang, Kun Lu, Cunmin Qu, Wei Hua, Jiana Li

**Affiliations:** 1 Chongqing Engineering Research Center for Rapeseed, College of Agronomy and Biotechnology and Academy of Agricultural Sciences, Southwest University, Chongqing 400716, China; drsunfujun@email.swu.edu.cn (F.S.); czy1988520@126.com (Z.C.); zqwvivi979@email.swu.edu.cn (Q.Z.); winyuan@email.swu.edu.cn (Y.W.); hr1996@email.swu.edu.cn (R.H.); ssl7159@email.swu.edu.cn (S.S.); cs19960301@email.swu.edu.cn (S.C.); nwyin80@swu.edu.cu (N.Y.); tys1998@email.swu.edu.cn (Y.T.); yliang@swu.edu.cn (Y.L.); drlukun@swu.edu.cn (K.L.); 2 Key Laboratory of Biology and Genetic Improvement of Oil Crops, Oil Crops Research Institute of the Chinese Academy of Agricultural Sciences, Ministry of Agriculture and Rural Affairs, Wuhan 430062, China; 3 Academy of Agricultural Sciences, Southwest University, Chongqing 400715, China; 4 Engineering Research Center of South Upland Agriculture, Ministry of Education, Southwest University, Chongqing 400715, China

**Keywords:** *TIFY* gene family, *Brassiceae* species, *Brassica napus*, evolutionary pattern, expression profiles, heavy metal stress

## Abstract

The TIFY gene family plays important roles in various plant biological processes and responses to stress and hormones. The chromosome-level genome of the *Brassiceae* species has been released, but knowledge concerning the TIFY family is lacking in the *Brassiceae* species. The current study performed a bioinformatics analysis on the TIFY family comparing three diploid (*B. rapa*, *B. nigra*, and *B. oleracea*) and two derived allotetraploid species (*B. juncea*, and *B. napus*). A total of 237 putative TIFY proteins were identified from five *Brassiceae* species, and classified into ten subfamilies (six JAZ types, one PPD type, two TIFY types, and one ZML type) based on their phylogenetic relationships with *TIFY* proteins in *A*. *thaliana* and *Brassiceae* species. Duplication and synteny analysis revealed that segmental and tandem duplications led to the expansion of the *TIFY* family genes during the process of polyploidization, and most of these *TIFY* family genes (*TIFY*s) were subjected to purifying selection after duplication based on Ka/Ks values. The spatial and temporal expression patterns indicated that different groups of *BnaTIFYs* have distinct spatiotemporal expression patterns under normal conditions and heavy metal stresses. Most of the JAZIII subfamily members were highest in all tissues, but JAZ subfamily members were strongly induced by heavy metal stresses. *BnaTIFY34*, *BnaTIFY59*, *BnaTIFY21* and *BnaTIFY68* were significantly upregulated mostly under As^3+^ and Cd^2+^ treatment, indicating that they could be actively induced by heavy metal stress. Our results may contribute to further exploration of *TIFY*s, and provided valuable information for further studies of *TIFY*s in plant tolerance to heavy metal stress.

## 1. Introduction

The *TIFY* gene family contains a conserved TIFY domain originally known as the Zinc-finger (ZIM) [1]. The TIFY domain of approximately 36 amino acids recognizes as the core motif TIF[F/Y]XG (where X represents any amino acid) [2]. Previous reports showed that members of the *TIFY* family could be divided into four subfamilies, namely ZML (ZIM/ZIM-like), PPD (PEAPOD), JAZ (jasmonate-ZIM-domain) and TIFY, based on their conserved protein domains [2,3]. The ZIM and ZIM-like (ZML) proteins, together forming the ZML subfamily, contain both a C2C2-GATA zinc-finger DNA-binding domain and a CCT domain (CONSTANS, CO-like, TOC1). The PPD subfamily contains a PPD domain at the N-terminus and a modified Jas domain at the PY motif is missing. The Jasmonate ZIM-domain (JAZ) subfamily contains a conserved Jas motif of approximately 27 amino acids near the C-terminus in addition to the TIFY domain [3,4].

The Jas domain has a similarity to the N-terminal portion of the CCT domain, with the characteristic motif SLX2FX2KRX2-RX5PY, which is necessary for interaction with other proteins, including the CORONATINE INSENSITIVE 1 (COI1) [5], the basic helix-loop helix (bHLH) transcription factors (e.g., MYC2, MYC3, MYC4, and MYC5), and MYB transcription factors (MYB21, MYB24, MYB75, and GLABRA1), through to the JA signaling pathway [5,6,7]. Among them, the *COI1* is the primary JA receptor [8,9], and MYC2, is an important transcriptional activator of responses to JA [10], as the link between COI1 and MYC2, JAZ proteins plays a key role in JA-mediated responses [11]. In *Taxus* spp., TcMYC2a can regulate taxon biosynthesis through binding with TcJAZ3 [10], while MYC3 and MYC4 could interact with the majority of JAZ proteins [12,13]. In *Arabidopsis thaliana*, the IIIe basic helix-loop-helix (bHLH) transcription factor MYC5 also acted as a target of JAZ repressors, functioning redundantly along with other IIIe bHLH factors, such as MYC2, MYC3, and MYC4 in the regulation of stamen development and seed production, and these IIIe bHLH factors interact with the MYB transcription factors (MYB21 and MYB24) to form a bHLH-MYB transcription complex, and cooperatively regulate stamen development [14]. In addition, the R2R3-MYB transcription factors, MYB21 and MYB24, were functional as direct targets of JAZs in regulating male fertility specifically [15]. However, JAZ proteins interacted with bHLH (e.g., Transparent Testa8, Glabra3, and Enhancer of Glabra3) and R2R3 MYB transcription factors (MYB75 and Glabra1) to repress JA-regulated anthocyanin accumulation and trichome initiation [16]; while both DELLA and JAZs could mediate the synergism between GA and JA signaling by interacting with the WD-repeat/bHLH/MYB complex [17]. To date, the roles of JA signaling in response to different environmental stimuli need to be further elucidated. 

In plants, *TIFY* family genes play significant roles in the regulation of diverse aspects of plant development, abiotic stresses and phytohormone treatments. For instance, *ZIM/TIFY1*, *TIFY8*, *AtTIFY4a* (PPD1) and *AtTIFY4b* (PPD2) were primarily observed in the process of plant growth and development [6,18]. In mango, expression patterns of the *TIFY* genes during fruit development and ripening indicated that they may be involved in development and ripening [19]. *TIFY11a* has been intensively investigated for its roles in salt tolerance in plants [20,21]. Extensive studies showed that most *TIFY* genes were widely involved in abiotic stress, such as drought, salt stress and cold stress in many species [20]. In rice, most *OsTIFY* genes were responsive to at least one type of abiotic stress, including drought, salt stress and cold stress [20]. Both rice and chickpea *JAZ* genes showed a certain level of macro (NPK) and micronutrients (Zn, Fe) deficiency, and the majority of them showed induction under K deficiency [22]. In addition, more efficient activation of *OsJAZ8* was accompanied by improved salt tolerance of the transgenic seedlings, and demonstrated the impact of temporal signatures of jasmonate signaling for stress tolerance [23]. In wheat (*Triticum aestivum*), *TIFY11a* was specifically induced after salt treatment. Transgenic lines overexpressing *TdTIFY11a* showed higher germination and growth rates under high salinity conditions, compared to wild type plants [21]. *GsJAZ2*, overexpressed in soybean and transgenic plants, could strongly increase their alkaline tolerance and confer JA insensitivity [24], while current basic knowledge of *TIFY* genes in heavy metal tolerance is still limited.

With its long history, the *Brassiceae* species not only provides more types of vegetables and edible vegetable oil, but is also known as an excellent evolutionary model for investigating the expansion of gene families [25]. Importantly, the *Brassiceae* species are considered to be heavy metal accumulators [26]. As such, we firstly performed a genome-wide identification and analysis of the *TIFY* family genes in *Brassiceae* species (*B. rapa*, *B. nigra*, and *B. oleracea*, *B. juncea*, and *B. napus*). We then analyzed the phylogenetic relationships, gene duplications, chromosome distribution, and motif compositions. Finally, we further investigated the expression patterns of *BnaTIFYs* in different tissues, and evaluated the specific roles of *BnaTIFY* gene family members in heavy metal induction. Our results provide valuable clues for the functional characterization of *TIFY* gene family, and lay a foundation for improving plant stress tolerance through the manipulation of *BnaTIFY* expression.

## 2. Results

### 2.1. Identification of TIFY Family Genes in Brassiceae Species

Using 18 *A*. *thaliana TIFY* family protein sequences as queries, a total of 237 TIFY family proteins were identified in *Brassiceae* species based on HMM profile and BLASTP analysis (Table S1), including 35 in *B. rapa*, 33 in *B. nigra*, 35 in *B. oleracea*, 64 in *B. juncea*, and 70 in *B. napus* (Table 1 and Table S2). We found that all identified *TIFYs* could be unevenly mapped across the whole chromosomes, except chromosome BjuA04, and chromosome BjuB03 contained the highest, with 10 *TIFY* genes (Table S2). The lengths of the TIFY protein sequences ranged from 112 (Bna*TIFY*08, *BnaTIFY12*, BnaTIFY13, BnaTIFY16, Bna*TIFY*49, and Bol*TIFY*10) to 1260 (Bju*TIFY*35) aa (amino acids) with an average length of 270 aa. The MW (molecular weights) of the TIFY proteins varied from 11.91 (Bna*TIFY*08, Bna*TIFY*13, and Bna*TIFY*16) to 137.89 (Bju*TIFY*35) kDa. Meanwhile, the pI (isoelectric point) was predicted as ranging from 4.26 (Bra*TIFY*31) to 11.43 (Bni*TIFY*22), but most of these were alkaline (197), with pI > 7.5, 36 were acidic with pIs ≤ 6.5, and others were neutral with 6.5 < pI < 7.5 (Table S2). Meanwhile, the predicted instability indexes showed that most TIFY proteins were unstable (Table S2). The subcellular localization of all *TIFYs* were only predicted and located on the nucleus, except for four *TIFYs* (*BjuTIFY05*, *BniTIFY07*, *BjuTIFY40*, and *BjuTIFY39*). Information on the detailed physical and chemical properties of all TIFYs and encoded proteins is listed in the Table S2

### 2.2. Phylogenetic Analysis of TIFY Genes in Brassiceae Species

**Figure 1:**
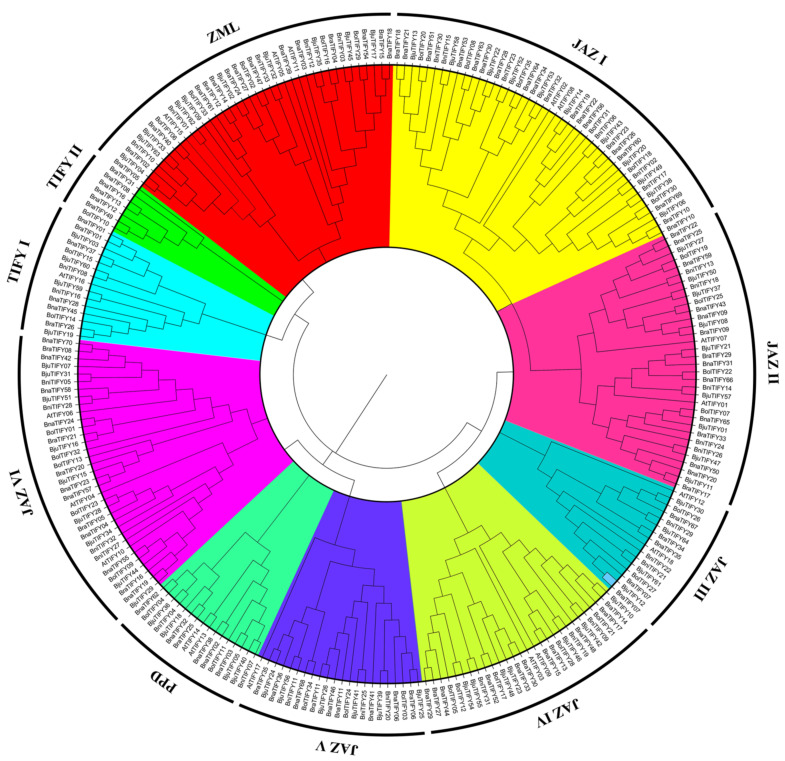
Phylogenetic tree of the *TIFY* family in *A. thaliana* and five *Brassiceae* species. The rooted neighbor-joining phylogenetic tree was constructed using MEGA7.0 and visualized using Figure Tree v1.4.2. The *TIFY*s were divided into ten subgroups (namely TIFY I, TIFY II, PPD, ZML and JAZ I-VI), which were indicated by different colors. Organism name and gene accession numbers are shown in Table S2.

To reveal the evolutionary relationships among the *TIFY* family genes, the NJ phylogenetic tree was constructed by the MEGA7.0 program (https://www.megasoft-ware.net/) based on the TIFY protein sequence alignments between *A. thaliana* and five *Brassiceae* species. As shown in Figure 1, 255 TIFY proteins were grouped into 10 clades, named JAZ I to JAZ VI, PPD, TIFY I, TIFY II, and ZML as defined by TIFY domain type [3], and members of the ten subfamilies were gathered separately. Of these *TIFY* members, 179 of the predicted *TIFY* genes contain both the TIFY domain and the Jas motif (namely JAZ I, JAZ II, JAZ III, JAZ IV, JAZ V, and JAZ VI), indicating that there is a close phylogenetic relationship within the JAZ subfamily. We found that 16 *TIFY* genes containing partial Jas (lack of a conserved PY) motif and additionally possessing a PPD domain in the N-terminal region were grouped into PPD subfamily; while 38 *TIFY* genes belong to the ZML subfamily with GATA-Zn finger domain, except for three *TIFY* genes (BjuTIFY02, BjuTIFY32, and BniTIFY33) (Table S1). Significant sequence similarity outside of the GATA-Zn finger domain might explain its presence in this subfamily. In addition, proteins classified into TIFY I and TIFY II were only contained in the TIFY domain, which included 15 and 7 TIFY proteins, respectively (Table S2). In general, the similar *TIFY* genes were divided into the same phylogenetic groups, revealing the parallel evolutionary relationship among the *A. thaliana*, the allotetraploids (*B. napus* and *B. juncea*) and their diploid progenitors (*B. rapa*, *B. oleracea*, and *B. nigra*). However, in group JAZ III, there were 16 JAZ subfamily proteins (two in *A. thaliana*, two in *B. rapa*, two in *B. oleracea*, three in *B. nigra*, three in *B. napus* and four in *B. juncea*). In group JAZ IV, there were 28 JAZ subfamily proteins (two in *A. thaliana*, four in *B. rapa*, five in *B. oleracea*, three in *B. nigra*, seven in *B. napus* and seven in *B. juncea*). These results suggest that *TIFY* family genes might undergo gene loss events in *Brassiceae* species during the process of polyploidization [27].

### 2.3. Chromosome Distributions of TIFY Family Genes in Brassiceae Species

**Figure 2:**
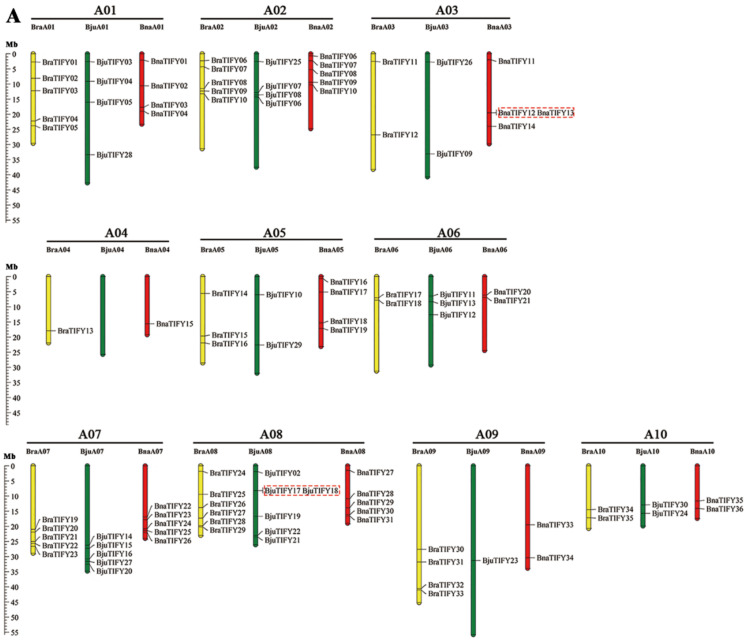

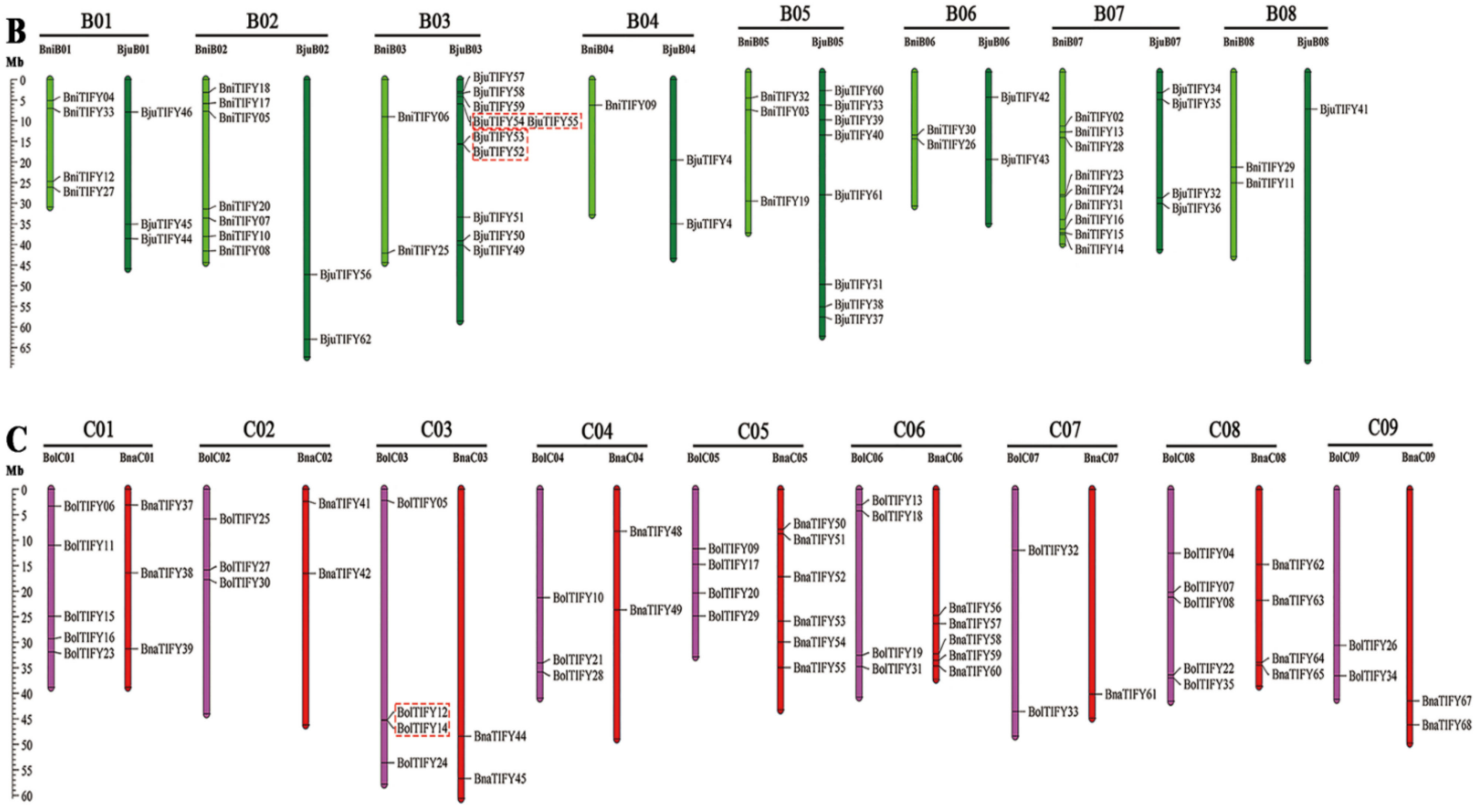
Chromosome distribution of *TIFYs* on sub-genome of *Brassiceae* species. (**A**) *TIFY* genes distributed on the A sub-genome in *B. rapa, B. juncea* and *B. napus.* (**B**) *TIFY* genes distributed on the B sub-genome in *B. nigra* and *B. juncea*. (**C**) *TIFY* genes distributed on the C sub-genome in *B.* *oleracea* and *B. napus*. The labels on the corresponding chromosomes indicate the name of the source organism and the sub-genome. The red rectangles represent the tandem duplications genes. The scales indicate the sizes of the various *Brassiceae* chromosomes (Mb). Bra, *B. rapa*; Bni, *B. nigra*; Bol, *B. oleracea*; Bju, *B. juncea* and Bna, *B. napus.* Detailed information on the genes located on the scaffold sequences is not shown here.

In this study, 220 *TIFY* genes were mapped to the corresponding chromosomes of Brassiceae species, including the A (99), B (62) and C (59) subgenomes, respectively (Figure 2), while 17 *TIFY* genes were mapped on the putative random chromosome (Table S2). As shown in Figure 2, all mapped *TIFY* genes were unevenly distributed on the chromosome, varying from 0 (BjuA04) and 1 (BraA04, BnaA04, BjuA09, BniB04, BjuB08, and BnaC07) to 10 (BjuB03). Meanwhile, five tandem duplication event regions were detected on chromosomes BnaA03 (1), BjuA08 (1), BjuB03 (2), and BolC03 (1), respectively. In addition, a hot spot of *TIFY* genes was generally more abundant at both ends of the chromosomes (Figure 2). Importantly, these similar *TIFY* genes generally were located in parallel physical positions among the same subgenome, indicating that extensive collinearity existed among the *TIFY* genes in *Brassiceae* species.

### 2.4. Synteny and Duplicated Gene Analysis of TIFY Genes in Brassiceae Species

**Figure 3:**
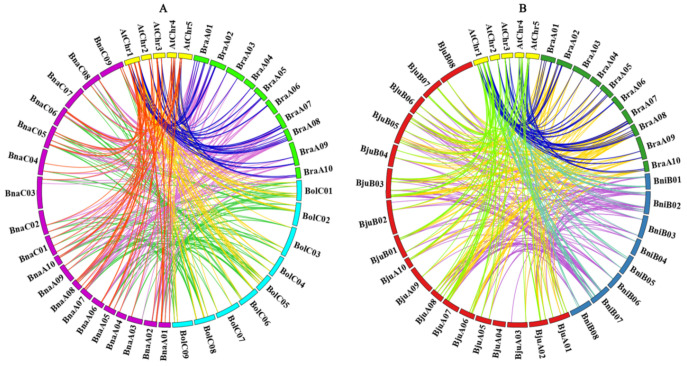
Synteny analysis of *TIFYs* between *A. thaliana* and five *Brassiceae* species. (**A**) Collinearity analysis of *TIFYs* among *A. thaliana*, *B. rapa*, *B. oleracea* and *B. napus*. (**B**) Collinearity analysis of *TIFYs* among *A. thaliana*, *B. rapa*, *B. nigra* and *B. juncea*. Different chromosomes are shown in different colors. Different gene pairs are represented by different colored lines.

Previous studies have shown that the protein-coding genes had large collinearity in *Brassiceae* species [28,29]. As shown in Figure 3, the retention or loss patterns of orthologous *TIFY* genes were displayed based on the syntenic relationships between *A. thaliana* and five *Brassiceae* species. Meanwhile, we also analyzed the comparative syntenic maps of the *A. thaliana*, the allotetraploids (*B. napus* and *B. juncea*) and their diploid progenitors (*B. rapa*, *B. oleracea*, and *B. nigra*) (Figure S1). Firstly, the number of *TIFY* genes in the allotetraploid *B. napus* (70) and *B. juncea* (64) is almost equal to that of the corresponding diploid *B. rapa* (35) and *B. oleracea* (35), and *B. rapa* (35) and *B. nigra* (33), indicating that these orthologous *TIFY* gene pairs have no divergence during polyploidization of *Brassiceae* species. Secondly, we performed synteny analysis of *TIFY* genes among *A. thaliana* and the five *Brassiceae* species. Herein, a total of 34 *BraTIFY* genes and 30 *BolTIFY* genes showed a syntenic relationship with the 17 *AtTIFY* genes and 59 *BnaTIFY* genes; 33 *BraTIFY* genes and 28 *BniTIFY* genes showed a syntenic relationship with the 17 *AtTIFY* genes and 61 *BjuTIFY* genes (Figure 3, Figure S1, Table S2). Furthermore, we identified 53, 46, 62, 109 and 97 orthologous *TIFY* genes between *AtTIFY* and *BraTIFY*, *AtTIFY* and *BolTIFY*, *AtTIFY* and *BnaTIFY*, *BraTIFY* and *BnaTIFY*, and *BolTIFY* and *BnaTIFY*, and 43, 78, 113, and 102 orthologous *TIFYs* between *AtTIFY* and *BniTIFY*, *AtTIFY* and *BjuTIFY*, *BraTIFY* and *BjuTIFY*, and *BniTIFY* and *BjuTIFY* (Table S3). These results show that the syntenic *TIFY* gene pairs were widely distributed on the genomes between *A. thaliana* and five *Brassiceae* species, and the *TIFY* genes had a high degree of retention among the five *Brassiceae* species during evolution.

In addition, the Ka, Ks, and Ka/Ks ratios for the *TIFY* gene pairs were separately calculated to predict the evolutionary constraints acting on the *TIFY* gene family between *A. thaliana* and five *Brassiceae* species. Results showed that Ka/Ks ratios were almost less than 1 between the orthologous *TIFY* gene pairs, except for *Bol034224* and *BnaA03g04250D*, *BraA09g057760.3C* and *BnaC08g37780D*, *BraA06g013190.3C* and *BnaA06g11690D*, *BraA02g020000.3C* and *BnaC02g20120D*, *BraA02g021020.3C* and *BnaA02g15990D*, *BraA07g029850.3C* and *BjuA027135* (Table S3). These results support that *TIFY* genes were subjected to purification selection during evolution.

### 2.5. Gene Structural and Conserved Motif Analysis of TIFY Family Genes in B. napus

**Figure 4:**
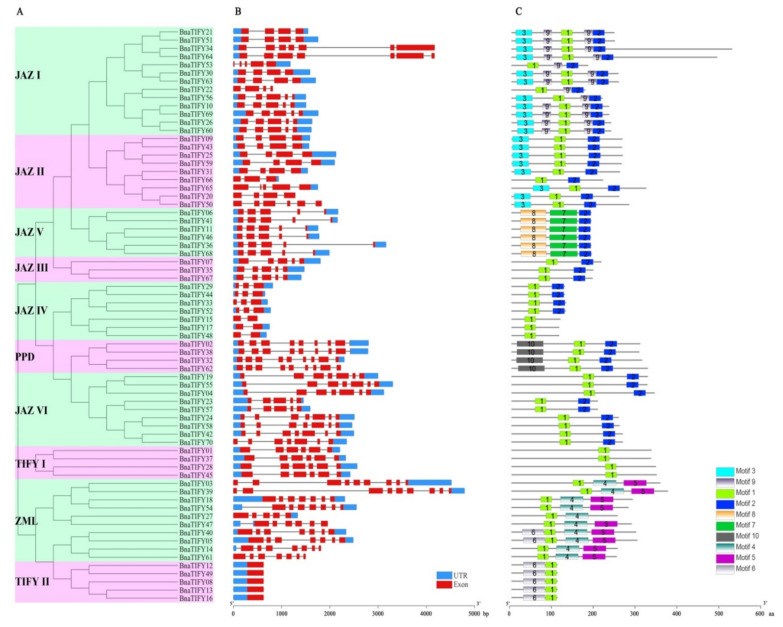
Phylogenetic relationships, gene structure and conserved motif of BnaTIFYs in *B. napus.* (**A**) Phylogenetic tree was constructed based on the 70 BnaTIFY protein sequences using MEGA7.0. (**B**) Gene structures analysis of *BnaTIFYs*. Red boxes represent exons and gray lines represent introns. The untranslated regions (UTRs) are indicated by blue boxes. The sizes of the exons and introns can be estimated using the scale at the bottom. (**C**) The conserved motif compositions of BnaTIFYs. Boxes with different colors indicate conserved motifs and listed in Figure S2.

We further explored the structural components of the *BnaTIFY* genes better understand their evolution in rapeseed based on phylogenetic analysis (Figure 4A). The number of exons in *BnaTIFY* genes varied from 1 (*BnaTIFY08*, *BnaTIFY12*, *BnaTIFY13*, *BnaTIFY16* and *BnaTIFY49*) to 9 (*BnaTIFY03* and *BnaTIFY39*), showing the consistency in the members of the same subfamily (Figure 4B). However, large divergences were found among *TIFY* genes in the different subfamily. For example, members of the JAZ IV and TIFY II subgroups contained fewer exons (1 to 3), while the highest exon numbers were found in the PPD subgroup, each member containing nine exons (Figure 4B). The results suggest that exon/intron structure further supports the phylogenetic structures of the *TIFY* genes.

As expected, the compositions of conserved motifs provide further confirmation of the evolutionary relationships among the BnaTIFY proteins (Figure 4C). In total, 10 motifs were identified and named Motif 1 to Motif 10 (Figure 4C). As mentioned above, the similar patterns of conserved motifs were listed in the same subfamily. Based on the Pfam domain search, all conserved motifs were identified, namely TIFY motif (motif 1 and motif 7), motif 2 (N-terminus of the Jas/CCT domain), motif 4 (CCT domain), motif 5 (GATA zinc-finger domain) motif 9 (C-terminus of the Jas domain), and motif 10 (PPD motif), respectively (Figure 4C, Figure S2). Apparently, motif 1 was the most frequent motif and existed in 64 BnaTIFY members, but motif 7 was specifically detected in the JAZ V subfamily. In addition, motif 4 and motif 5 were usually present in the ZML subfamily, while motif 2 were detected in the largest JAZ subfamily.

Altogether, the phylogenetic groups are consistent with the exon/intron structure and conserved motif compositions, indicating that the TIFY classifications are reliable in this study.

### 2.6. Expression Profiles of BnaTIFY Genes in B. napus

**Figure 5:**
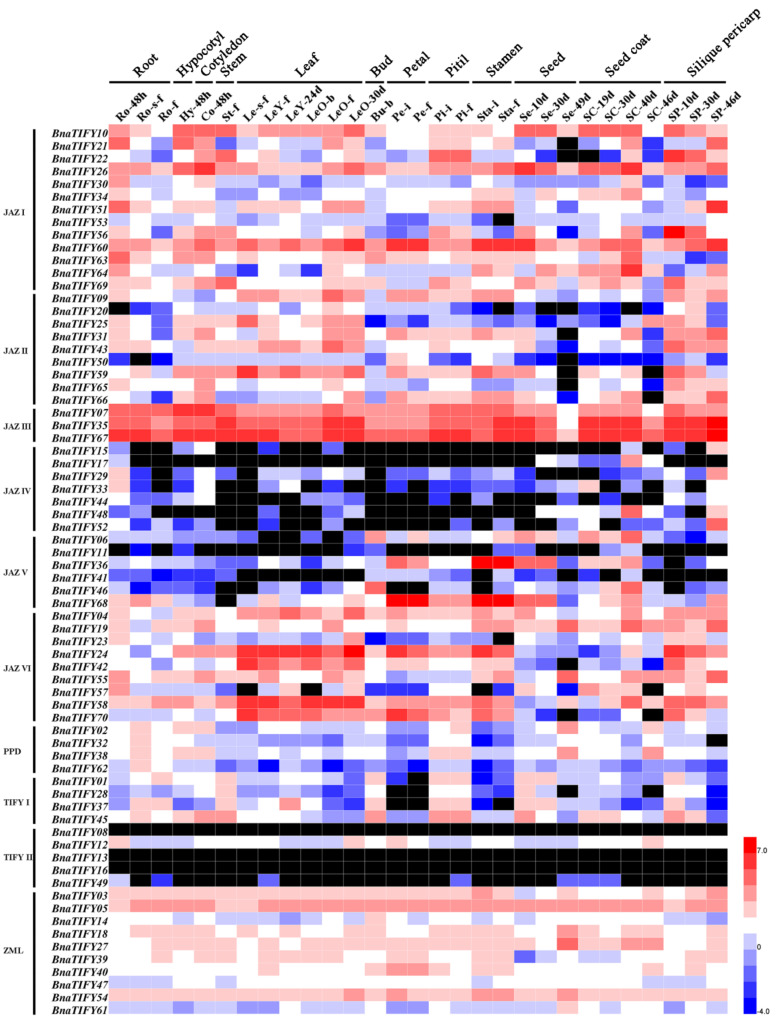
Expression patterns of all *BnaTIFYs* in different tissues and organs of cultivar ZS11. The heatmap was generated using the log_2_ expression levels (FPKM). The abbreviations of the different tissues of cultivar ZS11 are listed in Table S4. The bar represents the log_2_ expression levels (FPKM). Black boxes indicate that that FPKM values of *BnaTIFYs* are zero.

To explore the possible functions of Bna*TIFY* genes in rapeseed, we investigated the previously transcriptome sequencing datasets of B. napus cultivar Zhongshuang 11 (ZS11; BioProject ID PRJNA358784). The expression patterns of *BnaTIFY*s in various tissues, including the root, hypocotyl, cotyledon, stem, leaf, bud, petal, pistil, stamen, seed, seed coat, and silique pericarp (Table S4), were analyzed in this study. As shown in Figure 5, the *BnaTIFY*s displayed various differential expression levels among these detected tissues, indicating that Bna*TIFY*s were involved in multiple processes during plant growth and development. Among these, members in JAZ IV, JAZ V and TIFY II had near-to-low expression levels in the detected tissues, except for Bna*TIFY*12 in group TIFY II, while members in JAZ III showed the highest expression profiles in all the investigated tissues. Other *BnaTIFY* genes exhibited different expression patterns, suggesting that the *BnaTIFY* genes play important roles in all stages of growth and development.

### 2.7. Expression Profiles of BnaTIFY Genes in Response to As^3+^ and Cd^2+^ Treatments

**Figure 6:**
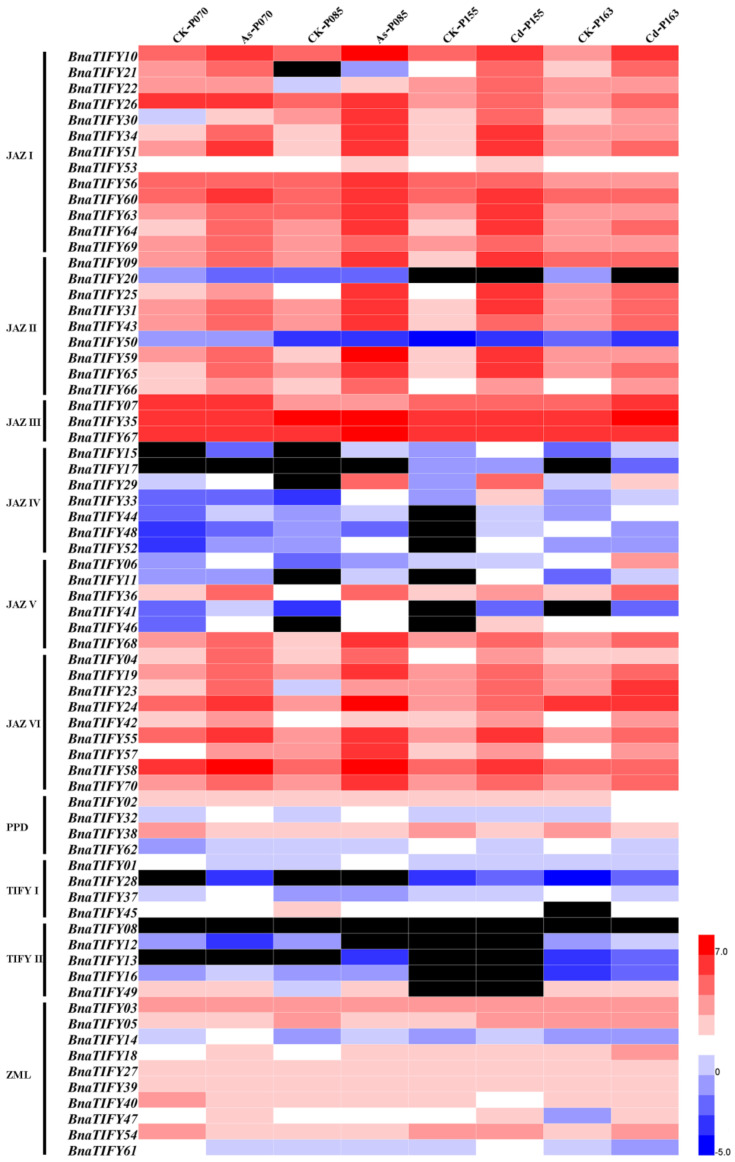
Expression profiles of the *BnaTIFYs* in response to As^3+^ and Cd^2+^ treatment. The expression profiles of each *BnaTIFY* were calculated as Log_2_ (FPKM values). Black boxes indicate that FPKM values of *BnaTIFYs* are zero (Table S5).

*TIFY* is a plant-specific protein family with a diversity of functions in plant development and responses to environmental stress [20,23,30]. To identify the response of *BnaTIFYs* to heavy metal stress, we investigated the expression patterns of *BnaTIFY* genes in rapeseed of seedling under 15 mg/L As^3+^ and 30 mg/L Cd^2+^ treatments. The expression levels of each *BnaTIFY* gene were determined by FPKM values (Table S5). Interestingly, the transcriptional levels of *BnaTIFY* genes were simultaneously induced by heavy metal treatment. As shown in Figure 6, most JAZ subfamily genes were strongly induced by heavy metal treatment, whereas others were not. 

**Figure 7:**
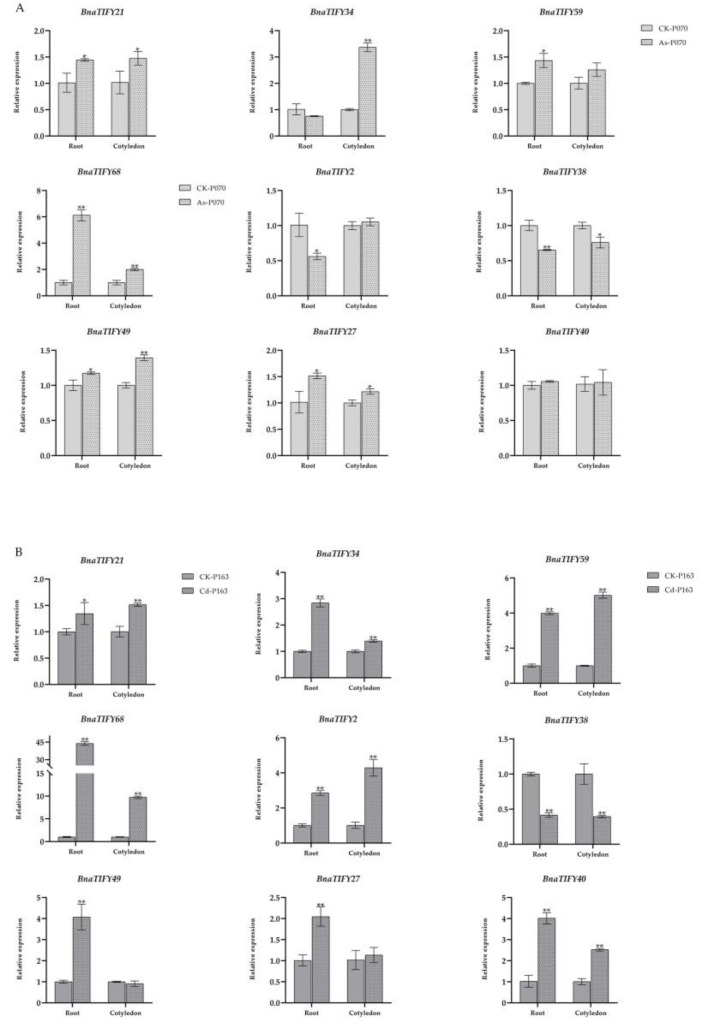
qRT-PCR analysis of 9 *BnaTIFYs* under As^3+^ (**A**) and Cd^2+^ (**B**) treatment. Data were normalized to *BnActin7* gene and vertical bars were obtained from three biological replicates. Statistically significant differences were analyzed by Student *t*-test. * *p* < 0.05; ** *p* < 0.01.

Among them, nine genes, *BnaTIFY21* and *BnaTIFY34* (JAZ I), *BnaTIFY59* (JAZ II), *BnaTIFY68* (JAZ V), *BnaTIFY02* and *BnaTIFY38* (PPD), *BnaTIFY49* (TIFY II), *BnaTIFY27* and *BnaTIFY40* (ZML), were selected to further validate by qRT-PCR analysis (Table S6). Overall, the expression patterns of the *BnaTIFYs* identified by qRT-PCR were similar to the patterns identified by RNA-seq (Figure 6 and Figure 7). Interestingly, *BnaTIFY49* were significantly upregulated under As^3+^ treatment (Figure 7A), *BnaTIFY2*, *BnaTIFY34* and *BnaTIFY59* were significantly upregulated under Cd^2+^ treatment (Figure 7B). We found that *BnaTIFY21* and *BnaTIFY68* were significantly highly expressed by As^3+^ and Cd^2+^ stress in cotyledons and roots; on the contrary, *BnaTIFY38* was significantly repressed, indicating that they have potentially function roles in heavy metal tolerance (Figure 7). In addition, they were also preferentially induced by heavy metal stress in different tissues, e.g., *BnaTIFY34* was significantly induced by As^3+^ in cotyledons, *BnaTIFY59* was significantly induced by As^3+^ stress in roots; *BnaTIFY27* and *BnaTIFY49* was significantly induced by Cd^2+^ stress in roots. These results suggest that these genes might have different functions in the plant response to As^3+^ and Cd^2+^ stress. Therefore, the specific mechanism via which *BnaTIFY* genes respond to the heavy metal stress in rapeseed requires further investigation.

In summary, *BnaTIFYs* have a wide variety of expression patterns in *B. napus*, most of which could be obviously induced by heavy metals, especially for the JAZ subfamily members. Our results provide important clues for further elucidation of the roles of *BnaTIFYs* in growth and development, and responding to heavy metal stress in *Brassiceae* species.

### 2.8. Cis-Element Analysis of BnTIFY Promotors 

Promoter *cis*-elements play pivotal roles in the transcriptional regulation of genes when plants are under biotic and abiotic conditions. As such, we carried out an analysis of transcription *cis*-regulatory elements in the 2000 bp regions upstream of *TIFY* genes transcription start sites in rapeseed, and *cis*-acting elements associated with abiotic and biotic stress, plant development and growth and phytohormones response were then identified (Table S6). In this study, most *BnaTIFY* genes possessed the ARE *cis*-elements (cis-acting regulatory element essential for the anaerobic induction), LTR *cis*-elements (cis-acting elements involved in low-temperature responsiveness) and MBS *cis*-elements (MYB binding sites involved in drought-inducibility). Moreover, 19 types of phytohormones responsive elements and 12 types of plant development and growth elements were predicted in the TIFY promoters (Figure S3). In addition, only *BnTIFY31, BnTIFY36* and *BnTIFY46* contained WUN-motif *cis*-elements (wound-responsive element) in their promotors (Figure S3, Table S6). These results confirm that the *TIFY* genes play a major role in stress resistance, plant biological processes and hormone signaling pathways.

## 3. Discussion

*TIFY* is the plant-specific family of putative transcription factors controlling a board range of developmental processes and various stress responses in plants [6,18,19,20,21,22,23,24]. Furthermore, the genome-wide analysis of *TIFY* family genes has been carried out extensively in different species [3,19,20,21,31,32,33,34,35]. However, information on the TIFY family gene is further investigated in *Brassiceae* species, which is helpful in understanding the resistance mechanisms to heavy metal. In the present study, 237 *TIFY* genes were identified within five *Brassiceae* species (*B.* *napus* and *B. juncea*, *B*. *rapa*, *B. oleracea*, and *B. nigra*). Moreover, the number of *TIFY* genes in the allotetraploid *B. napus* (70) and *B. juncea* (64) is almost equal to the sum of their diploid *B. rapa* (35) and *B. oleracea* (35), and *B. rapa* (35) and *B. nigra* (33) (Table S2). The results indicate that the number of *TIFY* genes showed variations among *Brassiceae* species. In addition, the results support the fact that *TIFY* genes varies in the complexity genome [34], with 18 in *Arabidopsis* [3], 20 in rice [20], 24 in poplar [33], 30 in maize [35], 34 in soybean [31] and 49 in wheat [21], and so on. Furthermore, these results also suppose that *TIFY* genes had greatly expanded in *Brassiceae* species, as well as that most family genes had undergone the numbers of the duplicated genes among them since their divergence from the common ancestor [36,37,38]. Correspondingly, we found that the highly strong purifying selection were predominately in most *TIFY* gene pairs based on the Ka/Ks values (Table S3).

Previously results showed that the *TIFY* family was divided into four major groups (ZML, TIFY, PPD and JAZ subfamilies) [3]. As anticipated, 255 identified TIFY proteins could be also classified into four major groups (ZML, TIFY, PPD and JAZ group) with ten subgroups (JAZ I to JAZ VI, PPD, TIFY I, TIFY II, and ZML) (Figure 1), indicating that *TIFY* genes had the functional conservation in the same group. In addition, the numbers in each subgroup varied from seven in TIFY II subgroup and 44 in JAZ I subgroup (Figure 1, Table S2), but further analysis of intron/exon patterns, motif structures showed the special subfamily characteristics with phylogenetic tree results in *Brassiceae* species (Figure 3). The results led us hypothesize that they may be highly conservative in their potential functions within the same groups. Accordingly, we could systematically predict the distinct function of the *TIFY* family members based on other syntenic orthologs in different species.

Numerous studies have revealed that *TIFY* family genes were widely responsible for plant growth and developmental processes in different tissues [18,19,22,32,39]. In the present study, we constructed a heatmap based on the relative expression levels of *TIFYs* in different *B. napus* ZS11 tissues. Our data revealed that the expression patterns of *TIFY* family genes showed obvious differences in the detected tissues (Figure 5), indicating that they may have differential functional roles across the whole of the development stages in rapeseed, of which the *TIFY* family genes in JAZ III subfamily had the highly expression levels in all tissues (Figure 5), indicating that they may be indispensability in rapeseed growth and development. Whereas JAZ VI subgroup members were specifically expressed in leaves (Figure 5), suggesting that these *TIFY* genes may play positive roles in leaves. Additionally, extensive evidence has reported the functional role of *TIFY* genes that are mainly responsible for various stress environments. For example, *OsJAZ* could be evidently induced by various abiotic stresses, including drought, salinity and low temperature, and *OsTIFY11a* could increase the stress tolerance in rice [20]. Similarly, *GaJAZ5* was found to improve drought resistance, and *GsTIFY10a* may play positive roles in plant tolerance to bicarbonate stress [40]. Recently, results showed that the *BnaJAZ* subfamily of genes could be predominantly induced by different abiotic/biotic stresses and hormones in rapeseed [41], which showed similar patterns in *B*. *rapa* [42] and *B*. *oleracea* [43]. In this study, our findings suppose that all *TIFY* family genes had a tight evolutionary and phylogenetic relationship (Figure 1 and Figure 4), and may share similar functions in the same subgroup. Therefore, the potential functional of *BnaTIFY* family genes that participate in plant stress tolerance can be predicted based on the previous description.

To date, heavy metal contamination has acted as one important constraint on crop productivity and quality [44]. Moreover, numerous evidence has supported that *Brassiceae* species have high tolerance to heavy metals [45], and are considered to be heavy metal accumulators [26]. Despite the fact that *TIFY* family genes are widely involved in environmental stress tolerance [20,40,41], few reports on *TIFY* family genes deal with heavy metal tolerance. In the current study, we analyzed the expression patterns of *BnaTIFYs* in response to As^3+^ and Cd^2+^ treatment. Obviously, most of the JAZ subgroup members were strongly induced by heavy metal stresses, while the PPD, *TIFY* and ZML subfamilies were slightly or not induced at all by infection with heavy metal stresses (Figure 6), suggesting that *BnaTIFYs* in the JAZ group predominantly the same ability as rapeseed in adapting to heavy metal stress. The results were further confirmed by qRT-PCR analysis, pointing to the reliability of our results. Importantly, we found that *BnaTIFY21* and *BnaTIFY68* were significantly more highly expressed by As^3+^ and Cd^2+^ stress in cotyledons and roots; on the contrary, *Bna*TIFY*38* was significantly repressed, suggesting that they played vital roles in responding to the heavy metal stress. Taken together, the present study systematically investigated the characterization of *TIFY* family genes in *Brassiceae* species, and is focused on the expression profiles of *BnaTIFY* family genes in various tissues and when responding to heavy metal stress, laying a solid foundation for further exploration of the roles of *TIFY* family genes in heavy metal tolerance.

## 4. Materials and Methods

### 4.1. TIFY Family Genes Identification and Annotation

To identify *TIFYs*, the genome and protein sequences of *A. thaliana*, and *Brassiceae* species were downloaded from the TAIR database (https://www.arabidopsis.org/), the Brassica Database (BRAD, http://brassicadb.cn) and the Genoscope database (https://www.genoscope.cns.fr/brassicanapus/ (30 August 2014)). Two approaches were performed to identify the members of the *TIFY* gene family. First, previously identified 18 At*TIFY* sequences from *A. thaliana* were used as queries to identify the candidate *TIFY* members in *Brassica* species via a BLASTp search (E value < 10^−5^). We then examined the conserved domain of the putative *TIFY* genes by the conserved domain database (CDD) of the National Center for Biotechnology Information (NCBI, https://www.ncbi.nlm.nih.gov/Structure/cdd) [46], and discarded the redundant genes without the *TIFY* domain. Second, the hidden Markov model (HMM) profiles of the *TIFY* domain (PF06200), Jas (PF09425) and CCT (PF06203) motifs were used to search putative *TIFY* genes in the *A. thaliana* and *Brassiceae* species using HMMER 3.0 software (http://hmmer.org/). The default parameters were adopted and the cutoff value was selected as an E value < 1e−6. In all, all of the putative *TIFY* genes were manually confirmed as having the *TIFY* domain, and used for further analysis. The number of amino acids, molecular weight (kDa), isoelectric point (pI) values, and stability index of each *TIFY* protein sequence were predicted using the ExPASy server (http://expasy.org). The subcellular locations were predicted using Plant-mPLoc in Cell-PLoc 2.0 (http://www.csbio.sjtu.edu.cn/bioinf/Cell-PLoc-2/ (30 May 2010)).

### 4.2. Sequence Analysis of TIFY Gene Family

Multiple alignments of *TIFY* proteins from *A. thaliana* and various *Brassica* species, including *B. rapa, B. oleracea*, *B. napus*, *B. juncea*, and *B. nigra*, were subjected to ClustalW software with default settings [47]. To illustrate the evolutionary relationships of the *TIFYs* in *Brassiceae* species, a neighbor-joining (NJ) phylogenetic tree was generated using the MEGA program (Tokyo Metropolitan University, Tokyo, Japan) with the Jones-Taylor-Thornton (JTT) + invariant sites (I) + Gamma (G) substitution model and a bootstrap test with 1000 replicates [48]. The phylogenetic trees were visualized using Figure Tree v1.4.2 (http://tree.bio.ed.ac.uk/software/figtree/ (31 July 2014)). The 2000 bp region upstream of the translation start sites of each *TIFY* gene was acquired from Brassica database (BRAD) as a promoter sequence, and the cis-elements were analyzed using the PlantCARE website (http://bioinformatics.psb.ugent.be/webtools/plantcare/html/ (30 September 2021)).

### 4.3. Conserved Motif Gene Structure Analysis of TIFY Gene Family

The conserved motifs in the *TIFY* family genes were analyzed using the online program Multiple Expectation Maximization for Motif Elucidation (MEME v4.12.0, http://memesuite.org/tools/meme (30 September 2021)) with the following parameters: number of repetitions any, maximum number of motifs 10; and optimum width of each motif between 6 and 300 residues [49]. The exon/intron structures of the *TIFYs* were analyzed using the Gene Structure Display Server 2.0 (GSDS 2.0, 30 September 2021), and which were visualized using TBtools software (https://github.com/CJ-Chen/TBtools (30 March 2020)) [50]. 

### 4.4. Chromosomal Locations, Tandem Duplication and Synteny of TIFY Gene Family

The position information of *TIFYs* were obtained from the *Brassiceae* genomic sequence annotation, and mapped to the corresponding chromosomes using MapChart v2.0 [51]. The gene duplication of *TIFYs* were analyzed by TBtools_JRE1.6 with default settings, and the tandemly duplicated genes of *TIFY* were identified according to their physical locations on individual chromosomes. Then the nonsynonymous (Ka) substitution rate and synonymous (Ks) replacement rate, and Ka/Ks ratio were used to estimate the selective pressure on the dataset. To well understand the synteny relationship of the orthologous *TIFY* genes, the syntenic analysis maps were generated using TBtools-Multiple Synteny Plotter with default parameters. Finally, the homologous genetic relationships of *TIFY* genes were displayed using TBtools-Amazing Super Circos program among different species [50].

### 4.5. Expression Profiles Analysis of TIFY Gene Family

Previously reported RNA-seq data (BioProject ID: PRJNA358784) were used to comprehensively investigate the spatio-temporal expression pattern of *BnaTIFYs* that participate in the throughout entire growth. The rapeseed cultivar “Zhongshuang 11 (ZS11)” were used as plant material. To investigate the expression pattern in response to heavy metal stress treatments, the expression profiles of *BnaTIFYs* were further analyzed using published RNA-seq dataset underAs^3+^ and Cd^2+^ stress [52]. The plant materials and treatments were as described previous [52]. All the relative expression levels of *TIFYs* were calculated and normalized as FPKM values (Fragments Per Kilobase of transcript per Million mapped reads) with TopHat and Cufflinks [53]. Finally, the heatmaps of *TIFYs* expression were created by TBtools [50] based on the normalized log2(FPKM).

### 4.6. Quantitative qRT-PCR Validation Analysis

The total RNA Extraction and qRT-PCR Analysis were performed as our described previous results [54]. In brief, total RNA was extracted from the samples using a DNAaway RNA Mini-prep Kit (Sangon Biotech, Shanghai, China), then the qualified total RNA was synthesized first-strand complementary DNA (cDNA) with an RNA PCR Kit (AMV) Ver. 3.0 (Takara, Dalian, China). We subsequently performed qRT-PCR analysis using SYBR Premix Ex Taq II (Takara, Dalian, China) on a Bio-Rad CFX96 Real Time System (Bio-Rad Laboratories, Hercules, CA, USA) as previously described [52]. Finally, *TIFY* gene expression levels were normalized using a reference gene *BnACTIN7* (EV116054) [55] via the 2^−∆∆Ct^ method. Three biological replicates were used in this study. The primers are listed in the Table S7.

## 5. Conclusions

In the present study, a total of 237 putative *TIFY* proteins with 10 groups were identified from five *Brassiceae* species based on their phylogenetic relationships with *TIFY* proteins in *A*. *thaliana* and *Brassiceae* species. Our duplication and synteny analysis revealed that segmental and tandem duplications led to the expansion of the *TIFY* gene family during the process of polyploidization, and most of these *TIFY* family genes were subjected to purifying selection after duplication. Finally, we explored the expression profiles of the *BnaTIFY* family genes in specific tissues, at various developmental stages, and in response to heavy metal stress via RNA-seq analysis and qRT-PCR. The spatial and temporal expression patterns indicated that JAZ III subfamily members were highest in all tissues, and JAZ subfamily members were strongly induced by heavy metal stresses. *BnaTIFY34*, *BnaTIFY59*, *BnaTIFY21* and *BnaTIFY68* were significantly upregulated mostly under As^3+^ and Cd^2+^ treatment, implied that they actively respond to expression under heavy mental stress. Our results may contribute to further exploration of the *TIFYs*, and provided valuable information for further studies of *TIFYs* in plant tolerance to heavy metal stress.

## Figures and Tables

**Table 1:** Statistics of *TIFY* family genes between *A*. *thaliana* and five *Brassiceae* species.

Group Name	*A. thaliana*	*B. rapa*	*B. nigra*	*B. oleracea*	*B. juncea*	*B. napus*
JAZ I	2	6	6	6	11	13
JAZ II	2	5	5	4	9	9
JAZ III	2	2	3	2	4	3
JAZ IV	2	4	3	5	7	7
JAZ V	1	3	3	3	6	6
JAZ VI	3	5	4	5	9	9
PPD	2	2	2	2	4	4
TIFY I	1	2	2	2	4	4
TIFY II	0	1	0	1	0	5
ZML	3	5	5	5	10	10
Total	18	35	33	35	64	70

## Data Availability

RNA-seq of *B. napus* variety Zhongshuang 11 (ZS11) in distinct tissues at different developmental stages are available in the NCBI Sequence Read Archive (SRA) database (BioProject ID: PRJNA358784). All other datasets supporting the results of this article are included within the article and its Additional files.

## Supplementary Materials

The following are available online at https://www.mdpi.com/article/10.3390/plants11050667/s1, Figure S1: Collinearity analysis of *TIFY* family genes among *A. thaliana* and five *Brassiceae* species, Figure S2: Protein motifs identified in different *B*. *napus TIFY* proteins through MEME motif searching, Figure S3: Predicted ***cis***-elements in *BnTIFY* promoters. Table S1: Pfam results of TIFY family genes identified from *A*. *thaliana* and five *Brassiceae* species, Table S2: Features of *TIFY* family genes identified from *A*. t*haliana* and five *Brassiceae* species, Table S3: The orthologous *TIFY* gene pairs among *A*. *thaliana* and five *Brassiceae* species, Table S4: *B. napus* ZS11 tissues and organs used in this study, Table S5: The relative expression values of *BnaTIFYs* by RNA-Seq analysis under As^3+^ and Cd^2+^ treatment, Table S6: The detailed information of cis-acting analysis among *BnTIFY* family gene promoters in *B*. *napus*, Table S7: Primers used to qRT-PCR analysis in this study.
