# Supplementary figures and images for: Genome-Wide Identification of the TIFY Gene Family in Brassiceae and Its Potential Association with Heavy Metal Stress in Rapeseed
Source: Plants (Basel). 2022 Feb 28;11(5):667. doi: 10.3390/plants11050667 (PMC8912736; doi:10.3390/plants11050667)

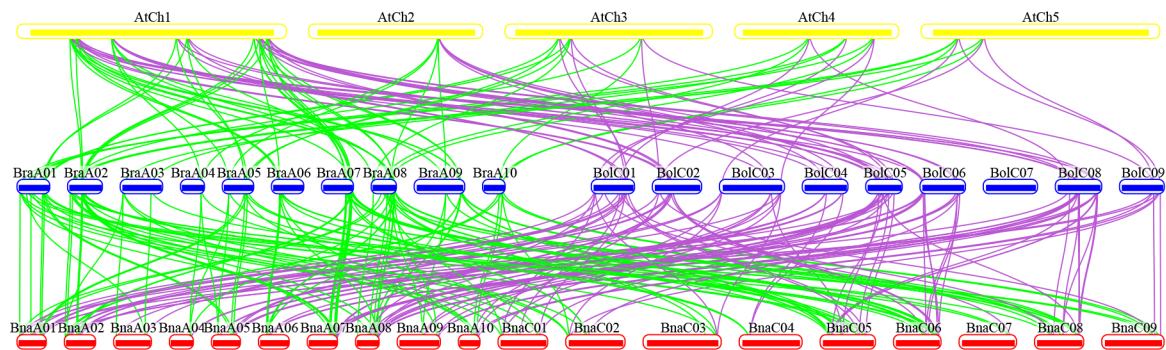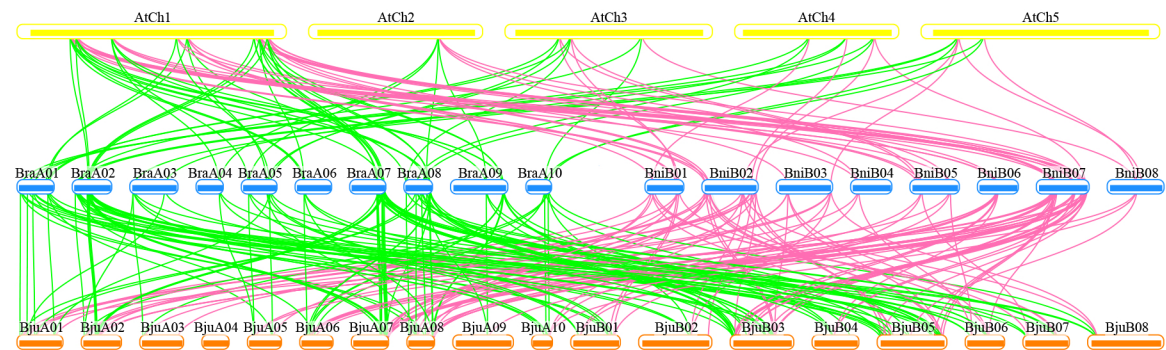

Supplement: Supplementary file 1 [file plants-11-00667-s001.zip › Figure S1. Collinearity analysis of TIFY family genes among A. thaliana and five Brassiceae species.pdf]

Motif 1

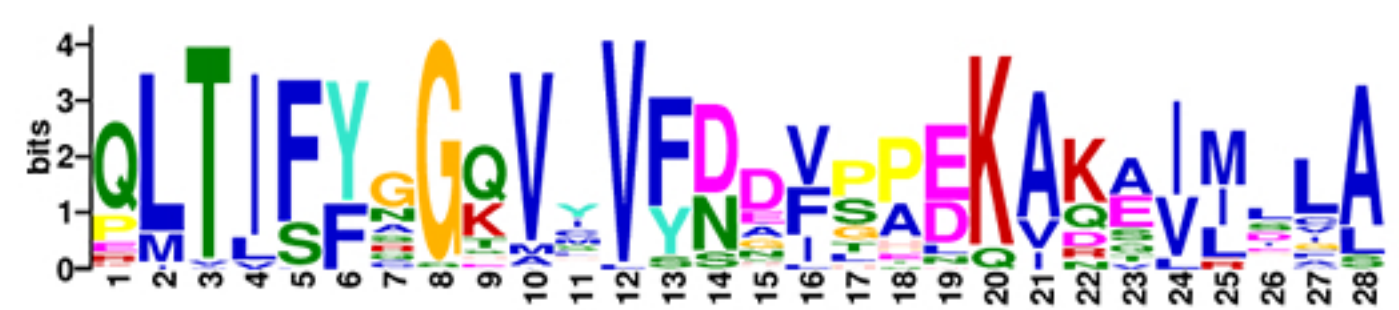

Motif 2

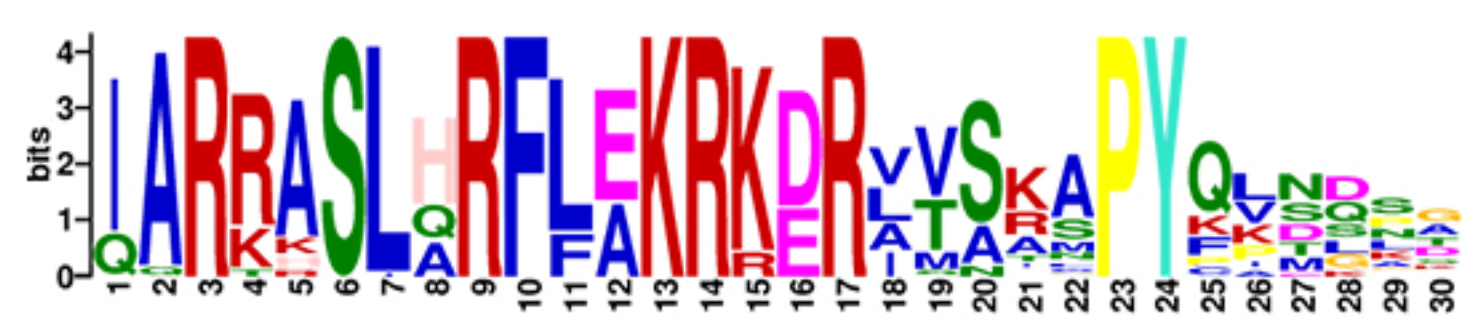

Motif 3

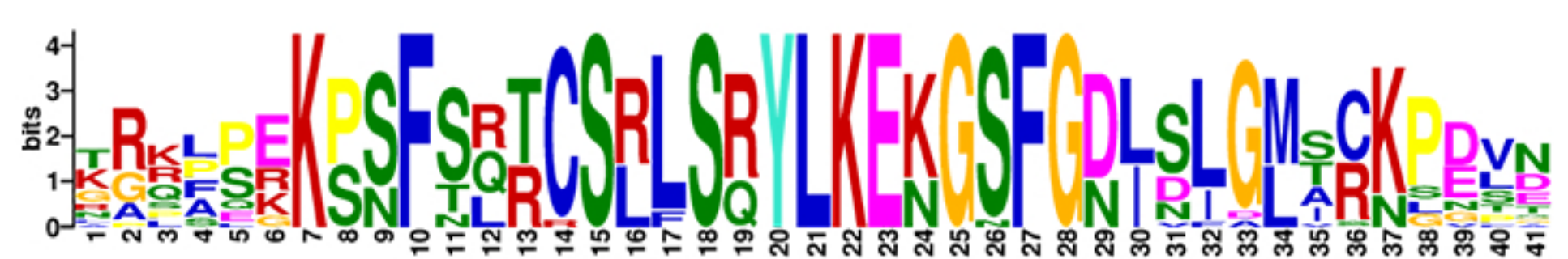

Motif 4

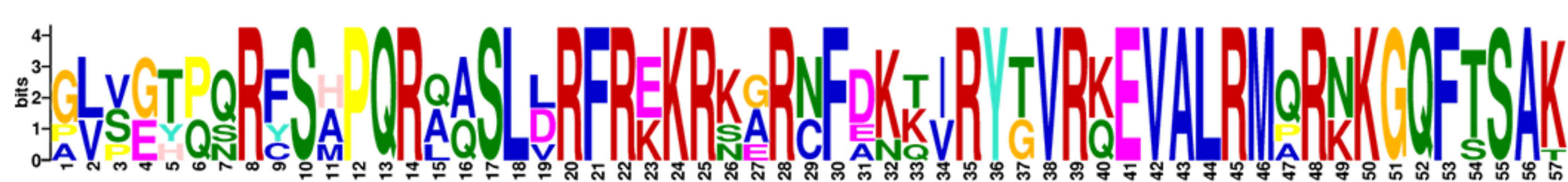

Motif 5

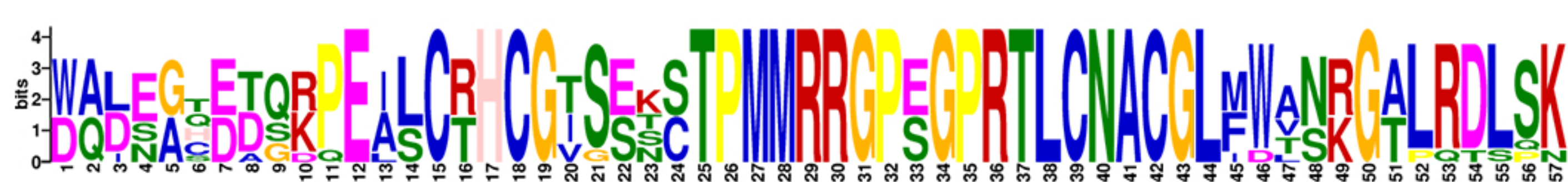

Motif 6

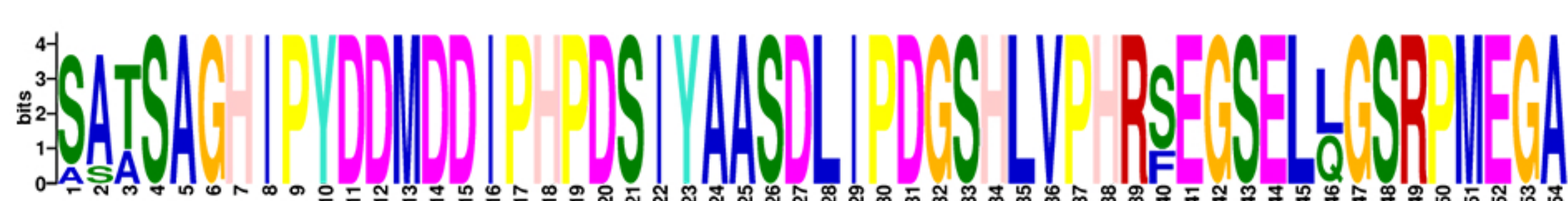

Motif 7

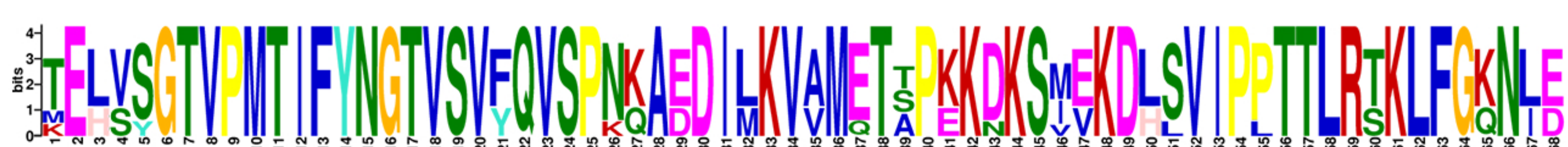

Motif 8

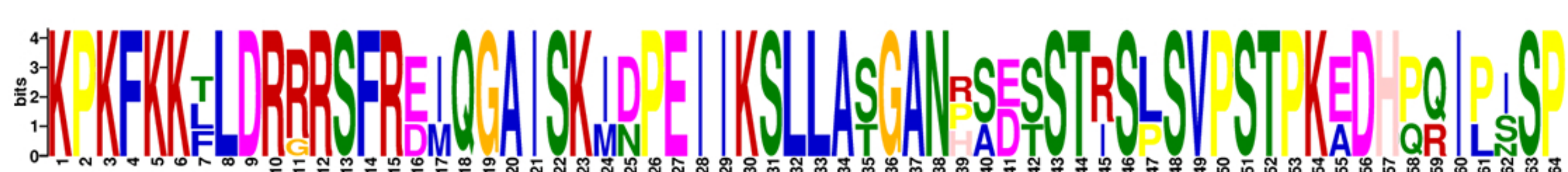

Motif 9

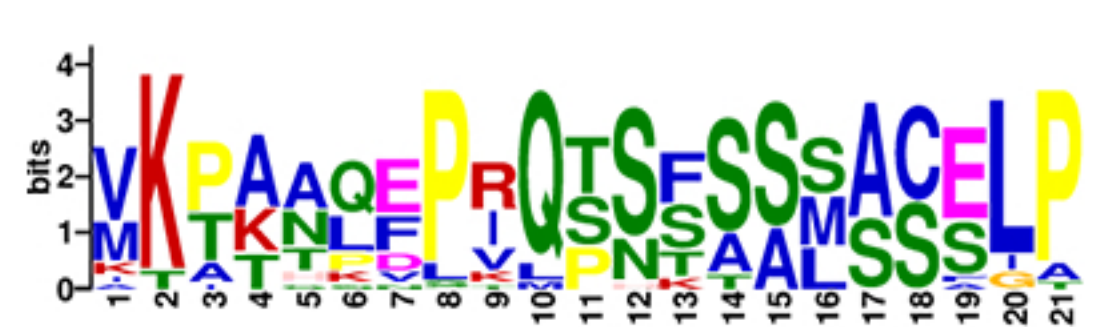

Motif 10

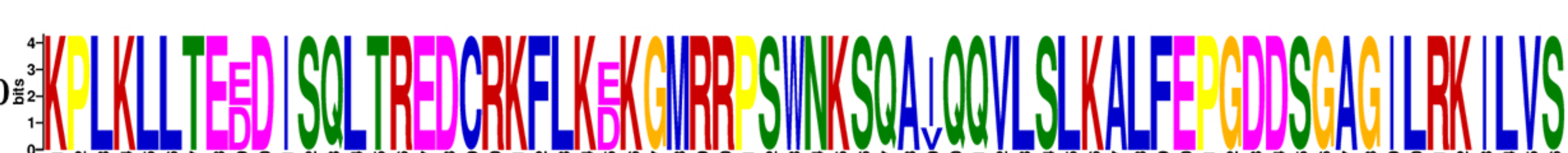

Supplement: Supplementary file 1 [file plants-11-00667-s001.zip › Figure S2. Protein motifs identified in different B. napus TIFY proteins through MEME motif searching.pdf]

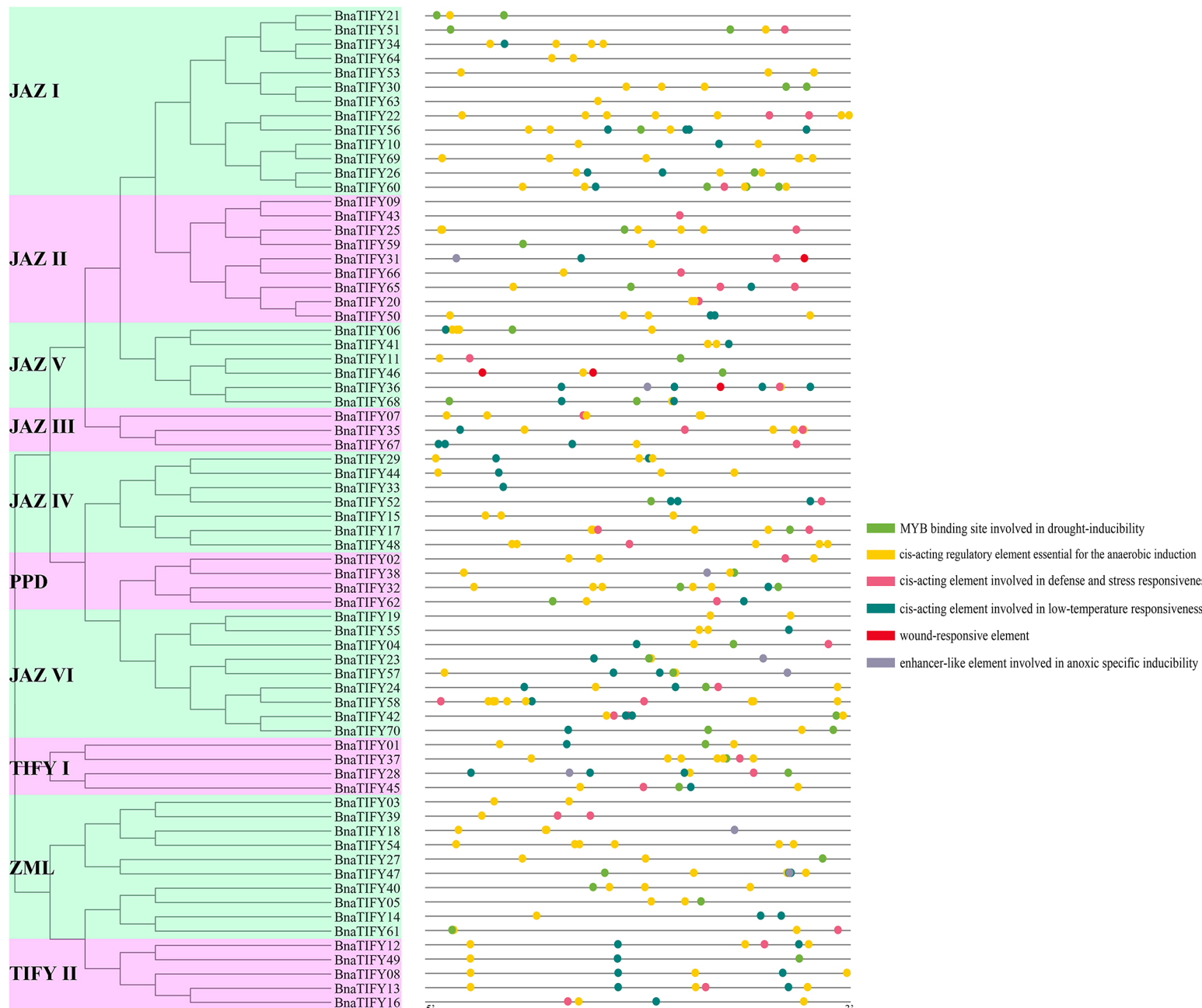

Supplement: Supplementary file 1 [file plants-11-00667-s001.zip › Figure S3. Predicted cis-elements in BnTIFY promoters.pdf]
